# Thermo-Electro-Chemo-Mechanical Coupled Modeling of Solid Oxide Fuel Cell with LSCF-GDC Composite Cathode

**DOI:** 10.3390/ijms24044137

**Published:** 2023-02-18

**Authors:** Weiqiang Cai, Qingrong Zheng, Jinliang Yuan, Wanneng Yu, Zibin Yin, Yu Wu, Zhonggang Zhang

**Affiliations:** 1 Marine Engineering Institute, Jimei University, Xiamen 361021, China; 2 Fujian Provincial Key Laboratory of Naval Architecture and Ocean Engineering, Xiamen 361021, China; 3 Faculty of Maritime and Transportation, Ningbo University, Ningbo 315832, China; 4 Shanghai Engineering Research Center of Hadal Science and Technology, College of Engineering Science and Technology, Shanghai Ocean University, Shanghai 201306, China

**Keywords:** solid oxide fuel cell, transport phenomena, thermal stress, methanol syngas, numerical analysis

## Abstract

Intricate relationships between transport phenomena, reaction mechanisms, and mechanical aspects likely affect the durability of solid oxide fuel cell (SOFC) stack. This study presents a modeling framework that combines thermo-electro-chemo models (including the methanol conversion process and the electrochemical reactions of the carbon monoxide as well as the hydrogen) and a contact thermo-mechanical model that considers the effective mechanical properties of composite electrode material. Detailed parametric studies are performed focusing on the inlet fuel species (hydrogen, methanol syngas) and flow arrangements (co-flow, counter-flow) under typical operating conditions (operating voltage 0.7 V), and performance indicators of the cell, such as the high-temperature zone, current density, and maximum thermal stress were discussed for parameter optimization. The simulated results show that the high temperature zone of the hydrogen-fueled SOFC is located at the central part of units 5, 6, and 7, and the maximum value is about 40 K higher than that of methanol syngas-fueled SOFC. The charge transfer reactions can occur throughout the cathode layer. The counter-flow improves the trend of the current density distribution of hydrogen-fueled SOFC, while the effect on the current density distribution of methanol syngas-fueled SOFC is small. The distribution characteristics of the stress field within SOFC are extremely complex, and the inhomogeneity of the stress field distribution can be effectively improved by feeding methanol syngas. The counter-flow improves the stress distribution state of the electrolyte layer of methanol syngas-fueled SOFC, and the maximum tensile stress value is reduced by about 37.7%.

## 1. Introduction

Solid oxide fuel cell (SOFC) is a highly efficient, environmentally friendly energy conversion system which has a promising application in the future [1,2]. Besides the advantages of SOFC that can be combined with gas turbines for co-generative applications, it can also utilize various fuels [3,4,5]. However, the commercialization of SOFC still has some challenges to overcome. High-temperature conditions (typical temperature 1073–1273 K) have more stringent requirements on the sealing and insulation of the system, which increases the cost of the system [6,7]. In addition, the electrode materials are prone to react with each other under high-temperature operating conditions, and the thermal stress caused by the temperature difference can also cause damage to the cell structure [8,9].

To address these problems, the appropriate lowering of the SOFC operating temperature has become a developmental trend [10,11]. Due to its high electronic conductivity, high oxygen ion conductivity, and high oxygen surface exchange coefficient, La_0.6_Sr_0.4_Co_0.2_Fe_0.8_O_3−δ_ (LSCF) has been recently proposed as the alternative electrode material for intermedium-temperature SOFCs [2,12,13,14]. Zheng et al. [13] compared the performance and degradation of the same three-cell stack with an H_2_O/H_2_ ratio of 90/10 and found that La_0.6_Sr_0.4_CoO_3−δ_ (LSC) cell and LSCF cell had lower degradation rates than LaSrMnO_3_ (LSM) cell. However, Co-based perovskite materials are negatively affected by the drawback of the reaction with YSZ electrolyte during operation [15]. This drawback can be avoided by applying a Ce_0.9_Gd_0.1_O_1.95_ (GDC) barrier layer to prevent the interfacial reaction between the YSZ electrolyte and the Co-based perovskite cathode electrode [14]. In addition, since the ionic conductivity of LSCF is greatly affected by the temperature drop, it is common to make a composite cathode by mixing LSCF with GDC material [14]. However, the microstructure of porous electrodes is characterized by tortuosity, porosity, and permeability, which makes physical-chemical phenomena significantly complicated. Moreover, the SOFC stack assembly leads to extra contact stress problems between different components. It is substantially difficult to measure experimentally the complex transport phenomena and related physical quantities inside SOFCs. In this regard, numerical simulations have gradually become an important technique for studying SOFCs [16,17].

Lee et al. [18] established a single-channel mathematical model fueled with hydrogen to study the transport phenomena inside the SOFC (Ni-YSZ/YSZ/GDC/LSCF-GDC). Numerical results showed that high fuel utilization induces a non-uniform electrochemical reaction zone near the fuel inlet and a large gradient of ionic current density along the cell. To elucidate the electrochemical reaction mechanism, Bessler et al. [19] developed a detailed model that includes an elementary-kinetic multistep description of electrochemistry with the coupled physical representation of the electric potential steps and charges transfer reactions. Methanol is considered a potential candidate for alternative fuels in SOFC because of its low impurity content, ease of storage and transportation, and convenient carrier for syngas. Given the effects of molecular weights of gas species and Knudsen flow mechanism, the dusty gas model (modified Stefan-Maxwell equations incorporating Knudsen diffusion) is used to model multi-component diffusion in the porous electrodes [2,3,4]. Xu et al. [20] established a two-dimensional numerical model to describe the thermal behaviors of the tubular SOFC (Ni-YSZ/YSZ/YSZ-LSM) fueled by methanol syngas. It is noted that the stability of methanol syngas-fueled SOFC could be substantially influenced by supplying sufficient steam to the fuel stream. This is because the endothermic methanol decomposition or internal reforming reaction would cause the local cooling and result in significant temperature gradients, although the overpotential losses and the electrochemical reactions tend to generate heat [21]. As reported by Khan et al. [22], highly coupled fluid flow, chemical/electrochemical reactions, and heat/mass transfer processes complicate the temperature field inside the cell, which could induce thermal stress resulting from the temperature gradient and the mismatch of the TEC (thermal expansion coefficient). Research on thermal stress behaviors of SOFC with the typical Ni-YSZ/YSZ/YSZ-LSM configuration has been carried out with varying degrees of model complexity [1,23,24,25]. Xu et al. [24] have numerically examined the thermal stress of SOFC (Ni-YSZ/YSZ/LSM) and found that the functional buffer layers can affect the stress between different components and inhibit the extent of degradation. Furthermore, as reported by Liu et al. [26], the stress state in the different layers is not permanent due to the temperature-dependent TEC and creep. 

From the literature research above, it is clear that no detailed thermo-electro-chemo-mechanical analysis of SOFC with LSCF-GDC composite cathode fueled by methanol syngas was conducted. Given that simultaneous thermal/mechanical solid-gas interactions and thermal-electrochemical-chemical reactions occur between the porous electrode surface and the gas species inside the SOFC, a three-dimensional numerical model from the previous research [27] is extended to elucidate the physical-chemical phenomena occurring in a planar SOFC with LSCF-GDC composite cathode. This study presents a modeling framework that combines thermo-electro-chemo models (including the methanol conversion process and the H_2_-H_2_O and CO-CO_2_ electrochemical reactions) and a contact thermo-mechanical model that considers the effective mechanical properties of composite material. The mechanical model is based on a full-sized SOFC stack loaded with a temperature profile obtained through accurate thermo-electro-chemo modeling (radiant heat exchange is considered). 

## 2. Results and Discussion

### 2.1. The Effects of Inlet Fuel Species

The fuel species components calculated in this study are hydrogen fuel with a molar fraction ratio of 99% H_2_ and 1% H_2_O, and methanol syngas fuel [19] with a molar fraction ratio of 10% H_2_, 67.5% H_2_O, and 22.5% CH_3_OH, respectively.

**Figure 1:**
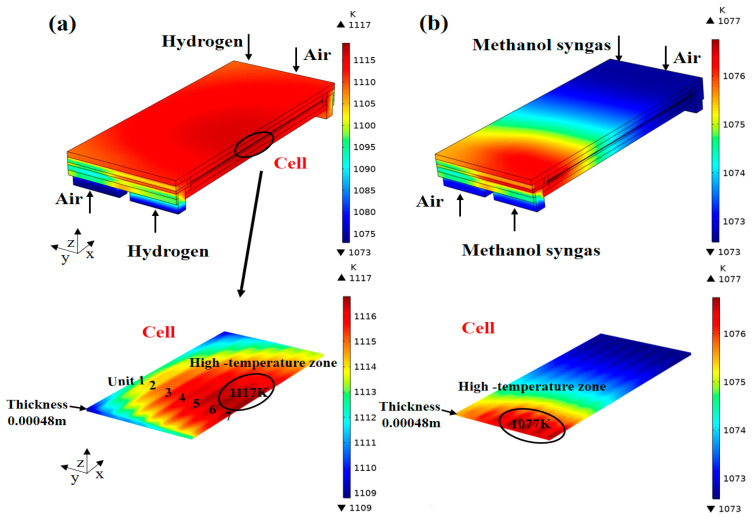
Temperature (K) distribution inside the SOFC for: (**a**) hydrogen fuel and (**b**) methanol syngas fuel.

The predicted temperature distribution in the SOFC fueled with different fuels is shown in Figure 1. It is found that the maximum temperature obtained in the methanol syngas-fueled SOFC is about 40 K lower than that of the hydrogen-fueled SOFC, and the temperature distribution is more uniform. In Figure 1a (hydrogen fuel), it is clear that the high-temperature zone (1117 K) of the cell is mainly concentrated in the central region of units 5, 6, and 7, and then gradually decreases from this region towards the surrounding walls. The heat is generated by the charged-species (oxygen ions and electrons) transport, activation polarization, and exothermic electrochemical reaction. The heat generated is predominantly conducted through metallic ribs owing to their large thermal conductivity [27], and the heat is in turn transported to the inlet gases carried by them. It is also noted that there is a significant temperature difference between the cell and the outside environment, which also causes some of the heat to be radiated from the outer walls through electromagnetic waves [23]. Thus, the thermal energy balance among heat generated, conduction, convection, and radiation induce inhomogeneities from the midst of the cell towards the surrounding walls. As for the methanol syngas-fueled SOFC (Figure 1b), the temperature decreases gradually along the x-direction, while the cooling effect of the MDR could be seen in the front region of units 5, 6, and 7, which lowers the maximum value (1077 K) of the cell temperature. It is found that the thermal neutral status can be attained in the latter part of the cell, indicating that the total heat generation rate is equal to the rate of heat consumption by the MDR, which is consistent with the published literature [20]. In addition, the temperature changes in the manifold are very small. Note that the large thermal expansion of the cells at the maximum temperature locations may result in non-uniform electrical contacts and mechanical loads imposing on the stack [18,28]. Therefore, given the location of the maximum temperature and its profile and increments, estimation of the mechanical behavior and thermal stress applied to the SOFC stack is critical for predicting the durability and performance of the materials, as will be shown in the following study.

**Figure 2:**
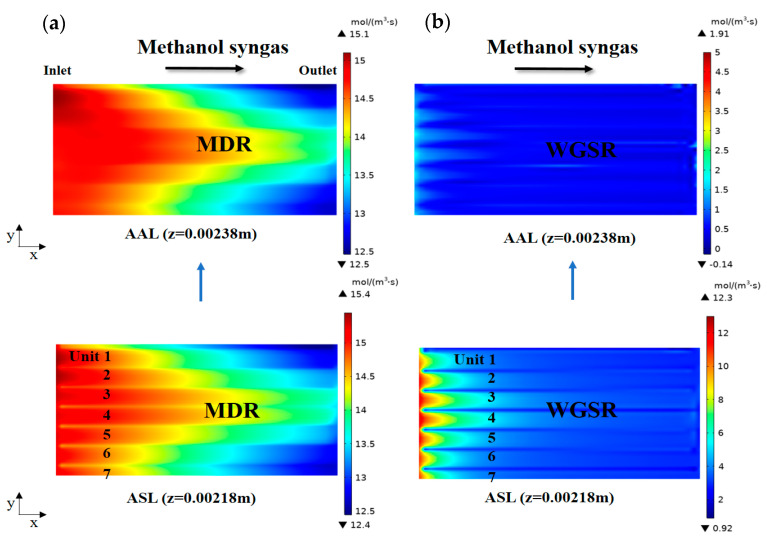
Reaction rate(mol·m^−3^·s^−1^) distributions in the plane (z = 0.00218, 0.00238 m) for: (**a**) hydrogen fuel and (**b**) methanol syngas fuel.

The predicted reaction rate (MDR, WGSR) of methanol syngas-fueled SOFC in the anode layer is shown in Figure 2. It is found that the general trend of the MDR reaction rate decreases gradually along the x-direction. The main factors that affect the MDR reaction rate are the heat required for the reaction and the supply of reactants involved in the reaction. From the temperature distribution law of methanol syngas-fueled SOFC above, it is clear that the anode layer near the manifold inlet has a higher temperature. This is due to the high initial concentration of H_2_ in the region, which leads to more heat release from electrochemical reactions. Similarly, the methanol concentration is higher in this region because of the proximity to the manifold inlet, where methanol can be supplemented in a timely manner. However, along the direction of the fuel flow, the concentration of the reaction gas is continuously depleted and the reaction rate gradually decreases. Different from the MDR reaction rate, the WGSR reaction rate becomes negative values in the AAL (x-y plane, z = 0.00238 m). This is because the CO-CO_2_ electrochemical reaction in the AAL continuously consumes a large amount of CO, which drives the WGSR in this region to perform the inverse reaction to reach equilibrium and the reaction rate thus becomes negative. In addition, the permeability of AAL is smaller than that of ASL [26], which also leads to a smaller CO concentration in AAL. It can be seen that, in the ASL (x-y plane, z = 0.00218 m), the reaction rate shows arcuate distribution regularity due to the transverse mass transfer.

**Figure 3:**
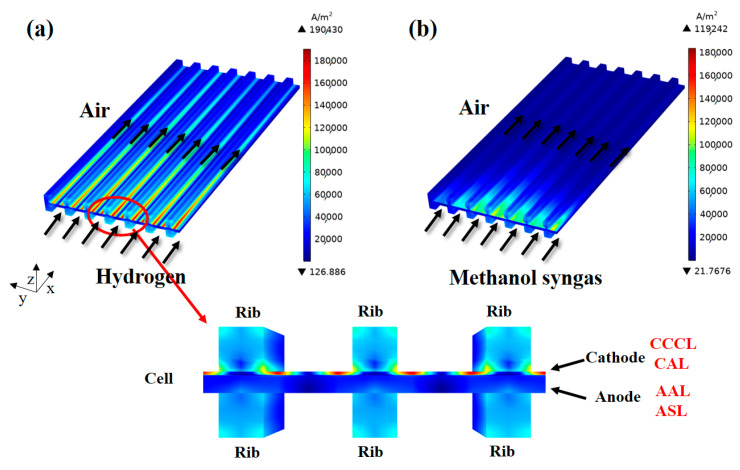
Current density (A·m^−2^) distribution inside the SOFC for: (**a**) hydrogen fuel and (**b**) methanol syngas fuel.

The magnitude and distribution of the current density inside the SOFC will directly affect the electrochemical performance and service life of the cell, while the experimentally measured current density in the I–V (current density-voltage) curve is only the average current density of the cell output at a certain point of operating voltage. The predicted current density distribution in the SOFC fueled with different fuels is shown in Figure 3. It is found that the current density distribution along the fuel flow direction (x-direction) is very uneven in the electrodes and the ribs. This is because the current density distribution is influenced by a combination of porous electrode structure (volume fraction of electronic conductor in the composite, reaction site area per unit volume, and pore size), fuel gas concentration, and operating conditions. Although the CO-CO_2_ electrochemical reaction and the H_2_-H_2_O electrochemical reaction occur simultaneously in the electrode layer of methanol syngas-fueled SOFC, the initial molar fraction of hydrogen (10%) in methanol syngas fuel is much smaller than that in hydrogen fuel (99%), and the initial molar fraction of CO is zero, resulting in the maximum current density of methanol syngas SOFC (119,242 A·m^−2^) being much smaller than that of hydrogen SOFC (190,430 A·m^−2^). Furthermore, from the enlargement view of the cell and ribs in Figure 3a, it can be found that the current density on the cathode side is much larger than that on the anode side, with the maximum value located at the interface between the cathode electrode and the ribs, while the minimum value appears at the anode electrode corresponding to the central region of the channels. One reason is that the reaction site area per unit volume of the cathode is much larger than that of the anode, and the thickness of the cathode layer is smaller. Another reason is that the channels are non-conductive and the electrons always transfer via the shortest path. Note that, the electrochemical reaction zone (in the z-direction) for the cathode (including CAL and CCCL) is significantly larger than that for the anode. Given that the cathode (CAL, LSCF-GDC; CCCL, LSCF) employs the mixed ionic-electronic conducting material LSCF [2], charge transfer reactions can occur throughout the cathode layer, which has not been demonstrated in most modeling studies of SOFCs composed of LSM cathodes assumed to be the cathode material [18,20,29].

Most researchers analyze the stress field of SOFC by comparing the first principal stress because the first principal stress is applied to the analysis of ceramic and metallic systems and can distinguish the maximum tensile and compressive stresses (positive and negative values of stress values represent tensile and compressive stresses) [17,23].

**Figure 4:**
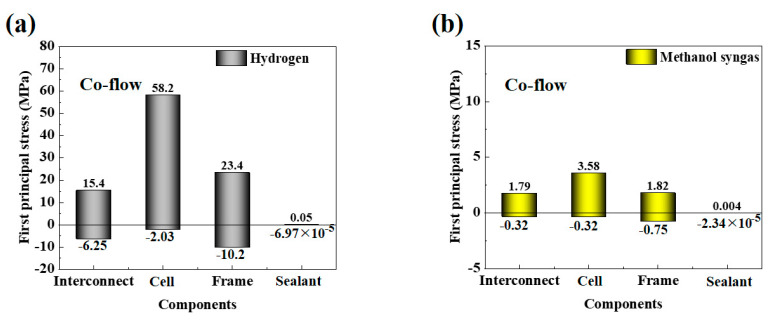
The variation range of thermal stress (MPa) of each component of the SOFC for: (**a**) hydrogen fuel and (**b**) methanol syngas fuel.

**Figure 5:**
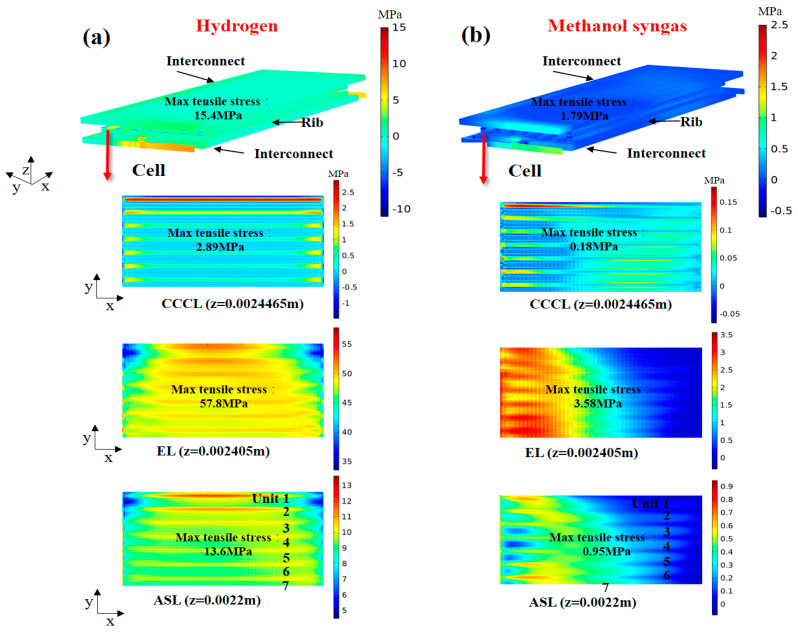
Thermal stress (MPa) distribution inside the SOFC for: (**a**) hydrogen fuel and (**b**) methanol syngas fuel.

The predicted variation range of the first principal stress of each component fueled with different fuels is shown in Figure 4. It is revealed that the range of thermal stress variation for the components fueled with hydrogen is larger than that fueled with methanol syngas. It is noted that the compression sealant (Flexitallic 866) has excellent material adaptability and can accommodate mismatched strains between components [22]. To further describe the details of internal processes occurring within the SOFC, the thermal stress distribution of the assembly components is also given, as shown in Figure 5. It is found that the distribution characteristics of the stress field are highly complex, and the stress extremes appear in the corner areas where the adjacent assembly components are in contact with each other. One reason is that the thermal expansion coefficient of the components does not match. Another reason is that the stack assembly leads to extra contact problem between different components. From Figure 5a (hydrogen fuel), it can also be seen that the electrolyte layer in the cell is subjected to the highest value of tensile stress. This is because the electrolyte in the cell has the lowest coefficient of thermal expansion, has the highest Young’s modulus, is more sensitive to external effects, and is susceptible to the mechanical constraints of adjacent components. As for the methane syngas fuel (Figure 5b), the stress field is more uniformly distributed and the electrolyte layer in the cell is subjected to the highest value of tensile stress. In addition, the thickness of the cathode current density collection layer is thin and subject to both tensile and compressive stresses, which is very likely to cause the material to curl, thus causing cracks in the cell.

### 2.2. The Effects of Flow Arrangements

**Figure 6:**
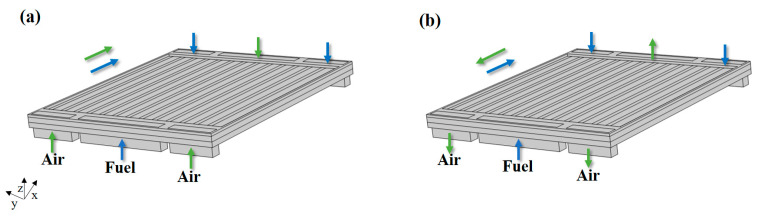
The flow arrangements for: (**a**) the co-flow and (**b**) the counter-flow.

To elucidate the layout of the co-flow and counter-flow arrangements (co-flow, the inlet air/fuel flows have the same direction; counter-flow, the inlet air/fuel flows have the opposite direction), a schematic sketch of a full-sized SOFC with different flow arrangements is given, as shown in Figure 6.

**Figure 7:**
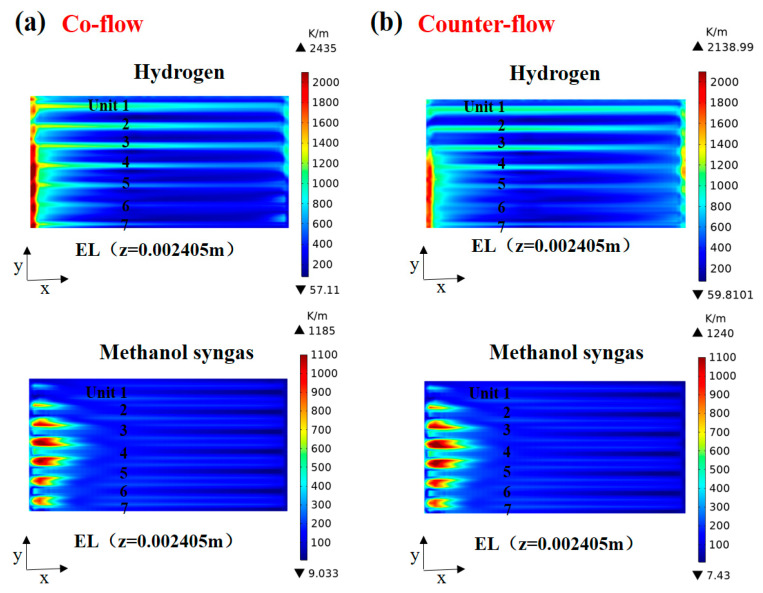
Temperature gradient (K/m) distribution in the plane (z = 0.002405 m) for: (**a**) co-flow and (**b**) counter-flow.

The predicted temperature gradient distribution in the plane (z = 0.002405 m) with different flow arrangements is shown in Figure 7. It is found, in Figure 7a (co-flow), that the high temperature gradient region of the hydrogen-fueled SOFC is concentrated on the edge side of the inlet area of units 5, 6, and 7, and the maximum temperature gradient is 2435 K/m. The temperature gradient near the cell wall is higher due to thermal radiation and thermal convection from the outer wall of the cell. The temperature gradient distribution of methanol syngas-fueled SOFC shows an arcuate similar to the reaction rate distribution. From Figure 7b (counter-flow), it is found that the high temperature gradient region of hydrogen fuel SOFC is concentrated in the inlet side of units 5, 6, and 7 and the outlet side of units 3 and 4, and the highest temperature gradient value is reduced by about 12.2%. The methanol syngas SOFC temperature gradient distribution is influenced slightly by the inlet gas method.

**Figure 8:**
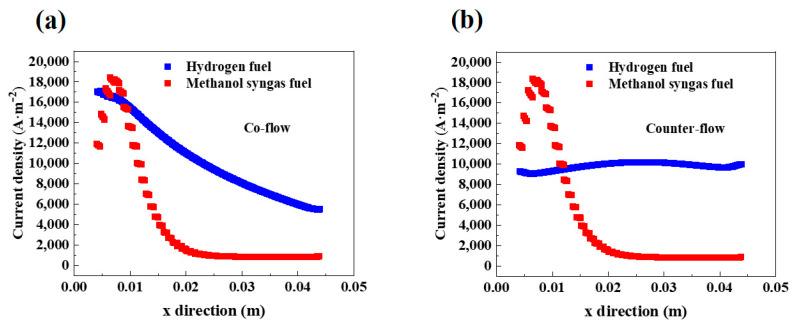
Current density(A·m^−2^) distribution along the x direction in the plane (z = 0.00238 m) at the position of unit 7 (y = 0 m) for: (**a**) co-flow and (**b**) counter-flow.

Figure 8 shows the distribution of current density along the middle section of AAL (y = 0 m, unit 7) in the forward *y*-axis with different flow arrangements. In Figure 8a (co-flow), the current density of hydrogen-fueled SOFC gradually decreases along the positive direction of the *x*-axis, while the current density of methanol syngas-fueled SOFC rises and then decreases along the positive direction of the *x*-axis, and reaches the maximum value near x = 0.01 m. This is because the initial content of hydrogen in the fuel is highest at the gas channel inlet, coupled with the strong MDR reaction to produce some CO. As the reaction proceeds, the H_2_-H_2_O electrochemical reaction and the CO-CO_2_ electrochemical reaction occur simultaneously, making the current density of methanol syngas fuel SOFC to reach the maximum, and then along the gas flow direction, the fuel gas content decreases, causing the current density to drop. From Figure 8b (counter-flow), it is revealed that the change of flow arrangements has a large effect on the trend of the current density distribution of hydrogen-fueled SOFC. This is because the initial hydrogen content at the anode channel inlet is high in the co-flow, while the counter-flow changes the inlet direction of air and hydrogen fuel, resulting in a lower air content at the anode channel inlet and weakening the electrochemical reaction between the anode channel inlet and the cathode channel inlet. Since the initial content of hydrogen in methanol syngas is very small and the initial content of CO is zero, the sensitivity of the H_2_-H_2_O electrochemical reaction and the CO-CO_2_ electrochemical reaction to the corresponding oxygen content is very low. Therefore, the change of flow arrangement has little effect on the trend of the current density distribution of methanol syngas SOFC.

**Figure 9:**
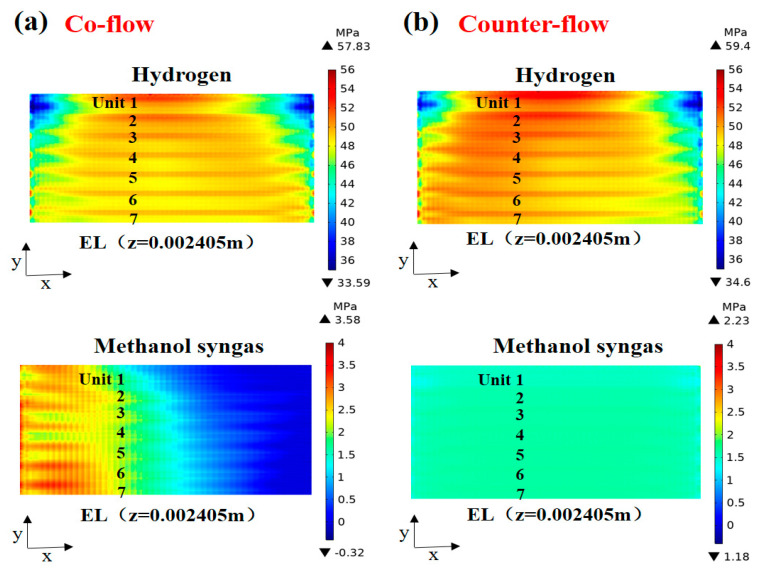
Thermal stress (MPa) distribution in the plane (z = 0.002405 m) for: (**a**) co-flow and (**b**) counter-flow.

The predicted thermal stress distribution in the plane (z = 0.002405 m) with different flow arrangements is shown in Figure 9. It is found, in Figure 9a (co-flow), that the high tensile stress region of the hydrogen-fueled SOFC is mainly concentrated in the central region of unit 1, while the high-stress region of the methanol syngas-fueled SOFC is mainly concentrated in the front part of the cell. This is consistent with the temperature field analysis above, as the thermal expansion of the electrolyte layer in the high-temperature region is larger than that in other regions with lower temperatures, enhancing the contact between the cell and the ribs, enhancing the contact between the cell and ribs. This results in a non-uniform mechanical load applied to the stack, leading to electrolyte degradation and cell warpage [24,28]. From Figure 9b (counter-flow), it is found that the stress state of SOFC of methanol syngas was changed from both tensile and compressive stresses to only tensile stresses, and the stress extremes were also reduced by 37.7%. Furthermore, it is noted that there is a significant stress gradient in the transverse direction (y-direction) of the electrode layer, which may induce a non-uniform contact between the components, leading to an unbalanced current distribution and the failure of compressive sealants [18]. This is because the rib directly corresponds to the electrode part constrained by the fixed stresses of the rib and the interconnector, while the channel cannot provide support.

## 3. Materials and Methods

### 3.1. Materials and Geometric Model

**Figure 10:**
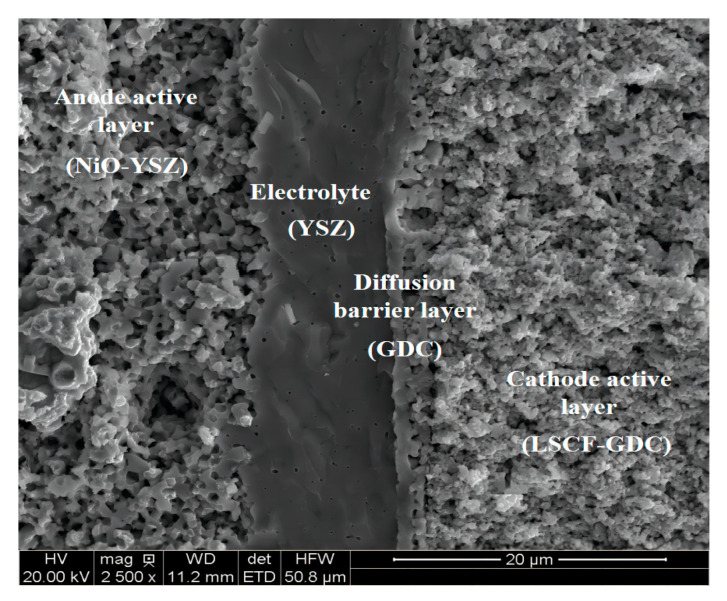
A SEM image of a tested cell cross-section, with (from left to right) AAL, EL, DBL, and CAL.

**Figure 11:**
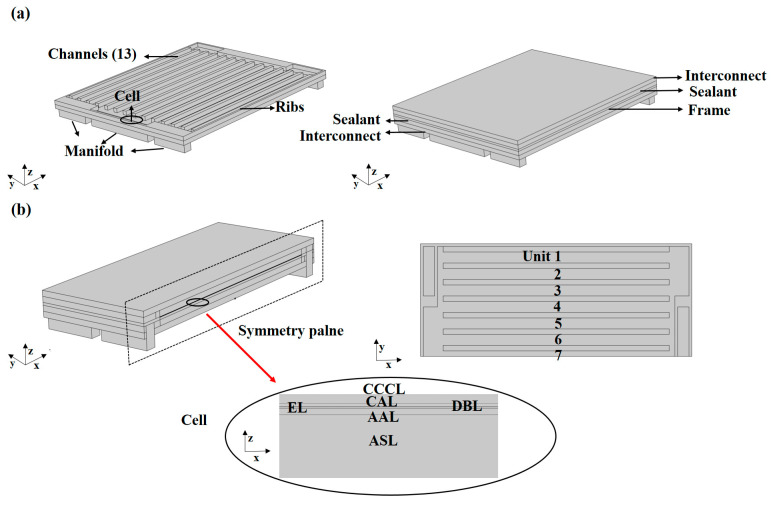
The geometry of anode-supported planar SOFC: (**a**) schematic of assembly system and (**b**) the computational domain.

The anode-supported planar single cell used in this study was manufactured by Ningbo SOFC-Man Energy Technology Co., Ltd, Ningbo, China. The tested cell had a 4 × 4 cm^2^ dimension (≥0.7 W/cm^2^) and consisted of a nickel-yttria-stabilized zirconia anode support layer (ASL), nickel-yttria-stabilized zirconia anode active layer (AAL), yttria-stabilized zirconia electrolyte (EL), Ce_0.8_Gd_0.2_O_2_ diffusion barrier layer (DBL), La_0.6_Sr_0.4_Co_0.2_Fe_0.8_O_3−δ_-Ce_0.8_Gd_0.2_O_2_ composite cathode active layer (CAL), and La_0.6_Sr_0.4_Co_0.2_Fe_0.8_O_3−δ_ cathode current collecting layer (CCCL). Since the LSCF is a mixed electronic and ionic conductor, the H_2_-H_2_O and CO-CO_2_ electrochemical reactions in the cathode layer occur at the interface of gas pore and LSCF. Given triple phase boundary percolation and its width, a reaction site area per electrode volume can be inferred, which is used in this study. The reaction site area of the CCCL (LSCF) is 4.88 μm^−1^, and that of composite CAL (LSCF-GDC) is 2.44 μm^−1^, both of which were measured by focused ion beam-scanning electron microscopy [18]. Figure 10 presents the scanning electron microscopy (SEM) micrograph of a cross-section of the tested cell. The corresponding experimental setup on which the physical model is based is shown in Figure 11a. In this study, the SOFC was divided into 6.5 units, and the unit closest to the edge of the SOFC was defined as unit 1, and so on thereafter. The SOFC interconnects and ribs are Crofer 22 APU stainless steel. The sealing material is Flexitallic 866. As the planar SOFC is symmetric, only the left half of the SOFC is selected as the simulation domain, as shown in Figure 11b. The geometrical parameters of the computational domain are shown in Table 1. 

### 3.2. Internal Reforming Reaction Mechanism

The air and methanol syngas is supplied to the cathode and anode manifolds, respectively. The methanol decomposition reaction (MDR) and water-gas shift reaction (WGSR) will occur in the porous nickel-based anode [20]. Although a small amount of methane might be produced by methanol decomposition, it is negligible according to previous experimental research [30]. MDR is shown in Equation (1), and WGSR is shown in Equation (2).

(1) $${\mathrm{CH}}_{3}\mathrm{OH}⇌{\mathrm{CO}+2H}_{2}{\;\Delta H}_{298K}^{0}=91\;\mathrm{kJ}/\mathrm{mol}$$

(2) $${\mathrm{CO}+H}_{2}O⇌{\mathrm{CO}}_{2}{+H}_{2}{\;\Delta H}_{298K}^{0}=-41.2\;\mathrm{kJ}/\mathrm{mol}$$

The reaction kinetic models (an Arrhenius form expression) from Mizsey et al. [31] for the MDR and from Haberman et al. [32] for the WGSR are used to calculate the reaction rates in this study. The equations for reaction rates (MDR, *R_m_*; WGSR, *R_s_*) are shown below:

(3) $$R_{m}=k_{mf}P_{CH_{3}OH}^{l}\left(1-\frac{P_{CO}^{l}{\left(P_{H_{2}}^{l}\right)}^{2}}{K_{pm}P_{CH_{3}OH}^{l}}\right)\left(\mathrm{mol}\cdot m^{-3}\cdot s^{-1}\right)$$

(4) $$K_{pm}=1.718\times 10^{14}exp\left(-\frac{95419}{RT}\right)\left({\mathrm{Pa}}^{2}\right)$$

(5) $$R_{s}=k_{sf}\left(P_{H_{2}O}^{l}P_{CO}^{l}-\frac{P_{H_{2}}^{l}P_{CO_{2}}^{l}}{K_{ps}}\right)\left(\mathrm{mol}\cdot m^{-3}\cdot s^{-1}\right)$$

(6) $$k_{sf}=0.0171exp\left(-\frac{103191}{RT}\right)\left(\mathrm{mol}\cdot m^{-3}\cdot {\mathrm{Pa}}^{-2}\cdot s^{-1}\right)$$

(7) $$K_{ps}=exp\left(-0.2935Z^{3}+0.6351Z^{2}+4.1788Z+0.3169\right)$$

(8) $$Z=\frac{1000}{T\left(K\right)}-1$$

where $P_{i}^{l}$ represents the pressure of species *i*, *k_mf_* and *k_sf_* represent the forward catalyzed reaction rate constants, and *K_pm_* and *K_ps_* represent the equilibrium constants.

The chemical reactions that occur inside the cell (absorption of heat, release of heat) result in a change in the corresponding heat source. The corresponding chemical heat changes (*Q_che_*) can be expressed as [20]:

(9) $$Q_{che}=R_{m}\Delta H_{m}+R_{s}\Delta H_{s}$$

(10) $$\Delta H_{m}=2h_{H_{2}}+h_{CO}-h_{CH_{3}OH}$$

(11) $$\Delta H_{s}=h_{CO_{2}}+h_{H_{2}}-h_{CO}-h_{H_{2}O}$$

where Δ*H_m_* and Δ*H_s_* are the enthalpy changes of MDR and WGSR, and *h_i_* is the enthalpy of formation of the species *i*.

### 3.3. Transfer Phenomena

The transfer processes that occur within the SOFC include the transfer of gas mass, momentum, charges, and energy. 

The mass transfer process refers to the transfer of gas components in manifolds, gas channels, and porous electrodes. The Stefan-Maxwell model can accurately describe the diffusion behavior of multi-component gas in a porous medium, but neglects the effects of the Knudsen flow mechanism. The dusty gas model considers the effects of molecular weights of gas species and the Knudsen diffusion [2,3,20]. The effective mass diffusion coefficient (*D_i,eff_*) of species *i* can be expressed as [20]:

(12) $$D_{i,eff}=\frac{\varepsilon }{\tau }\frac{\left(D_{i,gm}\cdot D_{i,k}\right)}{\left(D_{i,gm}+D_{i,k}\right)}$$

where *ε* is the porosity of the porous electrode, *τ* is the tortuosity, *D_i,gm_* is the gas diffusion coefficient, and *D_i,k_* is the Knudsen diffusion coefficient. 

The momentum transfer process refers to the transfer of momentum from the high-velocity fluid layer to the low-velocity fluid layer or the wall boundary layer. The Navier-Stokes equation is used to describe the momentum transfer process of the gas species inside the SOFC [23], which is expressed as:

(13) $$\nabla \left(\varepsilon \rho_{eff}UU\right)=-\varepsilon \nabla P+\nabla \left(\varepsilon \mu_{eff}\nabla U\right)+S_{d}$$

where the *ρ_eff_* and *μ_eff_* are the average density and average kinematic viscosity of the gas mixture, respectively, *U* is the velocity vector, and *S_d_* is the source term.

The charge transfer mainly includes the transfer of electrons in the conductor and the transfer of oxygen ions in the electrolyte material. The ion and electron conservation can be expressed as [27]:

(14) $$-\nabla \left(\sigma_{ion,eff}\nabla \phi_{e}\right)=A_{Ve}\times i$$

(15) $$-\nabla \left(\sigma_{elec,eff}\nabla \phi_{i}\right)=A_{Ve}\times i$$

where *σ_ion,eff_* and *σ_elec,eff_* are the ionic conductivity and electrical conductivity, respectively, *i* is the current density, and *A_ve_* is the specific surface area.

The energy transfer mainly includes enthalpy change caused by the diffusion of gas species, heat transfer between solid and gas phases, exothermic electrochemical reaction in the anode layer, and heat absorption by internal reforming reaction, etc. [4,18]. The local temperature equilibrium approach is applied to describe the energy transfer process in a porous medium, i.e., the temperature is assumed to be locally the same for the gases and solids [18]. The energy conservation can be expressed as:

(16) $$\nabla \left(\rho_{eff}c_{p,eff}UT\right)=\nabla \left(k_{eff}\nabla T-\overset{n}{\underset{i=1}{\sum }}m_{i}H_{i}\right)+S_{T}$$

where *c_p,eff_* is the effective specific heat capacity, *k_eff_* is the effective thermal conductivity, and *S_T_* is the heat source term.

### 3.4. Electrochemical Reaction Mechanism

It is reported that part of the CO in the syngas fuel also participates in the electrochemical reaction due to the equilibrium limitations of WGSR under high-temperature conditions [33]. Therefore, when hydrocarbons are supplied as fuel, both H_2_-H_2_O and CO-CO_2_ electrochemical reaction processes should be considered. In the anode, the electrochemical reaction is as shown in Equations (17) and (18).

(17) $$H_{2}{+O}^{2-}{=H}_{2}{O+2e}^{-}{\;\Delta H}_{298K}^{0}=-241\;\mathrm{kJ}/\mathrm{mol}$$

(18) $${\mathrm{CO}+O}^{2-}{=\mathrm{CO}}_{2}{+2e}^{-}{\;\Delta H}_{298K}^{0}=-283\;\mathrm{kJ}/\mathrm{mol}$$

In the cathode, the electrochemical reaction is shown in Equation (19).

(19) $$O_{2}{+4e}^{-}{=2O}^{2-}{\;\Delta H}_{298K}^{0}=-494.6\;\mathrm{kJ}/\mathrm{mol}$$

Considering the various polarization losses (activation polarization, ohmic polarization, and concentration difference polarization) that occur under operating conditions, the actual SOFC operating voltage *V_cell_* can be expressed as:

(20) $$V_{cell}=E-\eta_{act}-\eta_{conc}-\eta_{ohm}$$

where *E* is the electromotive force; *η_act_*, *η_conc_*, and *η_ohm_* are the activation overpotential, concentration overpotential, and ohmic overpotential, respectively. The *η_act_*, *η_conc_*, and *η_ohm_* can be calculated by Equations (21)–(23), respectively.

(21) $$\eta_{act}=\Phi_{elec}-\Phi_{ion}-\Phi_{eq}$$

(22) $$\eta_{conc,a}=\frac{RT}{2F}ln\left(\frac{y_{H_{2}}^{bulk}y_{H_{2}O}^{TPB}}{y_{H_{2}}^{TPB}y_{H_{2}O}^{bulk}}\right)+\frac{RT}{2F}ln\left(\frac{y_{CO}^{bulk}y_{CO_{2}}^{TPB}}{y_{H_{2}}^{TPB}y_{H_{2}O}^{bulk}}\right)\eta_{conc,c}=\frac{RT}{4F}ln\left(\frac{y_{O_{2}}^{TPB}}{y_{O_{2}}^{bulk}}\right)$$

(23) $$\eta_{ohm}=\underset{j}{\sum }i\frac{\delta_{j}}{\sigma_{j}}$$

where *Φ_elec_* and *Φ_ion_* are the potentials of electron and ionic, *Φ_eq_* is the equilibrium potential, *y^bulk^* and *y^TPB^* are the gas species mole fractions in the channel and electrode active layer, $\delta_{j}$ is the thickness of each electrode layer, and *σ_j_* is the conductivity of each electrode layer.

The polarization losses are strongly influenced by the electronic and ionic conductivity, *σ_elec_* (electronic conductivity) and *σ_ion_* (ionic conductivity) of given materials. The effective ionic conductivity and electronic conductivity at electrodes can be calculated by Equations (24) and (25), respectively [18].

(24) $$\sigma_{ion,eff}=\sigma_{ion}\left(1-\theta_{E,C}\right)\left(1-\varepsilon \right)$$

(25) $$\sigma_{elec,eff}=\sigma_{elec}\theta_{E,C}\left(1-\varepsilon \right)$$

where *θ_E,C_* is the volume fraction of electron conductor particles in the electrode material, which is 0.4 for the anode material Ni-YSZ and 0.5 for the cathode material LSCF-GDC. 

The electronic conductivity of the nickel metal is 7.15 × 10^6^ S·m^−1^, and that of LSCF mixed electron−ion conductor is 1 × 10^4^ S·m^−1^, and that of interconnect material is 8.7 × 10^5^ S·m^−1^ [18]. The ionic conductivity of the YSZ, GDC, and LSCF can be calculated by $\frac{1.64\times 10^{8}}{T}\exp\left(-\frac{9.46\times 10^{4}}{RT}\right)$, $\frac{3.16\times 10^{6}}{T}\exp\left(-\frac{5.33\times 10^{4}}{RT}\right)$, $\frac{2.57\times 10^{7}}{T}\exp\left(-\frac{8\times 10^{4}}{RT}\right)$, respectively [18].

### 3.5. Thermal Stress Model

The mechanical damage of the cell is considered to be directly related to the stress distribution, and the analysis of internal thermal stresses can predict the damage location. The thermal stress model assumed that ribs, interconnectors, sealants, and cell undergo linear (elastic) deformation when subjected to thermal loads, and mechanical theory conforms to Hooke’s law [23,24,25]. The stress-strain relationship of elastic material under thermal loading can be expressed as:

(26) $$\sigma =D\left(\xi -\xi_{th}-\xi_{0}\right)+\sigma_{0}=D\left(\xi -\alpha \left(T-T_{ref}\right)-\xi_{0}\right)+\sigma_{0}$$

where *σ* and *σ*_0_ are stress and initial stress, respectively; *D* is the elasticity matrix; ξ, $\xi_{th}$, and $\xi_{0}$ are total strain, thermal strain, and initial strain, respectively; *T* is the physical temperature for calculating the thermal stress, and *T_ref_* is the reference temperature for the free stress.

Compared with the traditional single-phase electrode, the dual-phase composite electrode material (nickel-yttria-stabilized zirconia, La_0.6_Sr_0.4_Co_0.2_Fe_0.8_O_3−δ_-Ce_0.8_Gd_0.2_O_2_) extends the triple phase boundaries within the electrode and improves the rate and efficiency of electrochemical reactions. Hsieh et al. [34] developed a modified unit-cell model which is widely used to predict the elastic and thermal constants of two-phase composites and was also used in this study. The parameters used in this thermal stress model are given in Table 2.

### 3.6. Solution Methodology and Boundary Conditions 

The three-dimensional mathematical model outlined above is developed and calculated by the COMSOL Multiphysics^®^ using the FEM (Finite Element Method). The PARDISO and GMRES were used for solving the fully-coupled nonlinear equations. The model was validated by comparing with experimental results in our previous work [26]. It should be noted that the same microstructures, materials, and operating conditions were considered for both numerical model and experiment.

The intermediate interface is set as a symmetry plane since only the left half of the cell is simulated in this study, and the SOFC is divided into 6.5 units, each of which includes gas channels, interconnects, ribs, and the corresponding cell, as shown in Figure 2b. The inlet gas flow rate of fuel and air is 6.68 × 10^−6^ m^3^·s^−1^ and 1.67 × 10^−5^ m^3^·s^−1^, respectively. Considering that the cell surface undergoes thermal radiation and thermal convection with the environment, the emissivity coefficient and convective heat transfer coefficient are 0.3 and 2 W∙m^−2^∙K^−1^, respectively [22]. Detailed input parameters and major boundary conditions of the SOFC model are summarized in Table 3.

The following assumptions are adopted in this model:

1. Steady state.
2. Gas species are deemed as ideal gases and gas flow is assumed to be laminar flow.
3. The reaction active sites are uniformly distributed, and the electronic and ionic conducting phases are continuous and homogeneous in the porous electrodes.
4. Ribs, interconnectors, sealants, cell, etc., are considered as isotropic materials and satisfy the isotropic Hooke’s law.

## 4. Conclusions

A comprehensive thermo-electro-chemo-mechanical coupled three-dimensional model is developed in this study. Detailed parametric studies were performed focusing on the inlet fuel species and flow arrangements under typical operating conditions (0.7 V) and distributions of temperature, current density, and thermal stress were discussed. The modeling approach can be applied to explore transport phenomena, reaction mechanisms, and mechanical behavior within the planar, tubular, and symmetrical SOFC.

The temperature field of SOFC is significantly influenced by the inlet fuel species. Due to the endothermic methanol decomposition reaction, the temperature distribution of methanol syngas-fueled SOFC is more uniform, and the maximum temperature value is only 1077 K. The counter-flow improves the inhomogeneity of the temperature gradient distribution inside the hydrogen-fueled SOFC and the maximum temperature gradient value is reduced by about 12.2%.

The charge transfer reactions can occur throughout the cathode layer and the high-current density region is mainly concentrated at the interface between the cathode electrodes, channels, and the ribs. The counter-flow improves the trend of the current density distribution of hydrogen-fueled SOFC, while the effect on the current density distribution of methanol syngas-fueled SOFC is small.

The distribution characteristics of the stress field within SOFC are extremely complex and the inhomogeneity of the stress field distribution can be effectively improved by feeding methanol syngas. The thermal expansion of the electrolyte layer in the high-temperature region is larger than that in other regions with lower temperatures, enhancing the contact between the cell and the ribs. The counter-flow improves the stress distribution state of the electrolyte layer of methanol syngas-fueled SOFC, and the tensile stress maximum value is reduced by about 37.7%.

## Figures and Tables

**Table 1:** Geometrical parameters of simulation domain.

Parameters	Values (m)	Parameters	Values (m)
Length/width of anode-supported SOFC (*x*/*y*-axis)	4 × 10^−2^/2 × 10^−2^	Length/width of manifold cross section (*x*/*y*-axis)	2 × 10^−3^/9 × 10^−3^
Thickness of interconnect (*z*-axis)	1 × 10^−3^	Thickness of ASL (*y*-axis)	3.65 × 10^−4^
Thickness of sealant (*z*-axis)	1 × 10^−3^	Thickness of AAL (*z*-axis)	3.5 × 10^−5^
Thickness of frame (*z*-axis)	4.8 × 10^−4^	Thickness of EL (*z*-axis)	1 × 10^−5^
Height/width of channel (*z*/*y*-axis)	1 × 10^−3^/2 × 10^−3^	Thickness of DBL (*z*-axis)	3 × 10^−6^ m
Height/width of rib (*z*/*y*-axis)	1 × 10^−3^/1 × 10^−3^	Thickness of CAL (*z*-axis)	1.7 × 10^−5^ m
Height of manifold (*z*-axis)	2 × 10^−3^	Thickness of CCCL (*z*-axis)	5 × 10^−5^ m

**Table 2:** The parameters used in this thermal stress model [23,28].

	Ni	NiO	YSZ	LSCF	GDC	Interconnect	Sealan
Young’s modulus *E* (GPa)	0.25	90	185	10	90	60	0.019
Poisson’s ratio *v*	0.3	0.34	0.313	0.32	0.32	0.3	0
TEC *α* (10^−6^ K^−1^)	16.2	13.0	10.5	26	12.63	15.5	15.5

**Table 3:** Input parameters and major boundary conditions of the SOFC model.

	Boundary Conditions	Temperature/K	Species	Ions	Electrons
Anode manifold inlet	Sccm: 200	1073	Fuel	None	None
Cathode manifold inlet	Sccm: 800	1073	Y_O2_ = 0.233, Y_N2_ = 0.727	None	None
Top of upper interconnect	Wall	Adiabatic	None	None	0 V
Bottom of lower interconnect	Wall	Adiabatic	None	None	0.7 V
Both sides of the cell	Wall	Adiabatic	None	None	None
Anode channel outlet	P_out_ = P_atm_ = 1 atm	Convection	Convection	None	None
cathode channel outlet	P_out_ = P_atm_ = 1 atm	Convection	Convection	None	None

## Data Availability

Not applicable.
